# Comparative evaluation of treatment of angular bone defect related to over-erupted tooth using guided tissue regeneration (GTR) followed by orthodontic intrusion (OI) versus OI followed by GTR: a controlled clinical trial

**DOI:** 10.1186/s12903-024-04036-9

**Published:** 2024-02-24

**Authors:** Rehab F. Ghouraba, Neveen Fakhry Abotaha, Sara Mohamed Ahmed Sagha

**Affiliations:** 1 Oral medicine, Periodontology, Oral Diagnosis and Radiology Department, Faculty of Dentistry, Tanta University, Tanta, Egypt; 2 Orthodontic Department, Faculty of Dentistry, Tanta University, El-Giesh St, Tanta, Gharbia Egypt

**Keywords:** Over-erupted tooth, Angular bone loss, Guided tissue regeneration, Orthodontic intrusion

## Abstract

**Background:**

Prematurity resulted from pathological migration of periodontally involved teeth with the loss of vertical stopping points between teeth, which can lead to teeth over eruption with dimensional changes favoring occlusal discrepancies. Therefore, evaluating and comparing the effect of guided tissue regeneration followed by orthodontic intrusion as opposed to orthodontic intrusion tracked by guided tissue regeneration in the treatment of an over-erupted tooth with angular bone loss.

**Methods:**

Twenty teeth in ten cases were selected with at least two teeth with vertical over-eruption and angular bone loss with the presence of their opposing. In group one, ten teeth over-erupted were treated by guided tissue regeneration followed by orthodontic intrusion, whereas, in group two, ten teeth over-erupted were treated by orthodontic intrusion followed by guided tissue regeneration. They were evaluated clinically for pocket depth, bleeding on probing, and tooth mobility. Radiographical evaluation assessed by cone beam computed tomography.

**Results:**

Clinically, there existed a statistically significant difference (P value ≤ 0.05) in favor of group one at six months post and in favor of group two at one year from re-evaluation regarding pocket depth and tooth mobility. Radiographically, in group one, there was a statistically significant improvement (*P* value ≤ 0.05) at six months post-guided tissue regeneration or orthodontic intrusion regarding defect depth and dimensional changes of the defect area, with a statistically significant difference (*P* value ≤ 0.05) in favor of group two at one year from re-evaluation phase regarding defect depth and defect area dimensional changes.

**Conclusion:**

There was a short-term improvement in group one, which deteriorated over a long period compared with group two, so it is preferable to start orthodontic intrusion before guided tissue regeneration.

## Introduction

Ortho-perio challenges present in patients with advanced horizontal bone loss [1] or hemiseptal defects (angular defects) of one- or two-wall osseous defects that are found around mesially tipped teeth or teeth that have supra-erupted [1, 2]. These kinds of bone loss are usually accompanied by occlusal prematurity due to pathological tooth migration leading to dimensional changes from the loss of vertical stopers between teeth [3, 4], in which modern therapeutic interventions are focused on effective cooperation and interaction between orthodontics and periodontics [5, 6]. Orthodontics and periodontics progress in harmony; in certain cases, orthodontic tooth movement improves periodontal integrity, while in other cases, periodontal treatment methods like phase one therapy and guided bone regeneration (GBR) to replace lost bone render orthodontic tooth movement easier [7]. So, the perplexing question of whether orthodontic treatment or periodontal therapy comes first in the management of ortho-perio difficulties remains. The number of adult orthodontic patients with periodontal issues has been greater than in the past due to the age-related increase in periodontal disease incidence [8]. Clinically, pathologic tooth migration appears as extrusion (over-eruption) with traumatizing occlusion has been described [7, 9–12]. As such, discrepancies in tooth location may favor plaque accumulation, complicate plaque control, traumatize the periodontal tissue, and impair aesthetics and function [13]. Vertical orthodontic tooth movement (intrusion) is not just to improve specific osseous deficiencies in patients with periodontitis, but also to remove the requirement for resective osseous surgery [7].

However, fixed orthodontic appliances could affect the health of the periodontium, especially with subgingival bands, leading to further inflammation and bone loss, which was confirmed histologically by Diedrich et al., [14]. Furthermore, active orthodontic treatment increases the risk of apical progression of tooth junctional epithelium with bone loss, tooth pronclination, rotation, and traumatic occlusion, all of which can lead to tooth loss [15]^,^ [16]. Therefore, this study was designed to assess and contrast the effects of guided tissue regeneration (GTR) followed by orthodontic intrusion (OI) with OI followed by GTR in the treatment of over-erupted teeth with angular bone loss on both the clinical and radiographic levels. In this study, the null hypothesis is that there is no appreciable variation between the two groups.

## Materials and methods

A controlled clinical trial was used to conduct this study. It was done at Tanta University’s Faculty of Dentistry in the departments of orthodontics and periodontology. Ten healthy participants in this study were between the ages of twenty and thirty-five. They were chosen from patients who visited outpatient periodontal and orthodontic clinics. Each patient had at least two teeth that had over-erupted in two different locations, each with opposing teeth.

The research for this study received approval from Tanta University’s Faculty of Dentistry Research Ethics Committee under code (#R-OMPDR-1-20-1). In accordance with the standards for human research approved by the Research Ethics Committee of the Faculty of Dentistry, Tanta University which follows the ethical guidelines outlined in the 1964 Helsinki Declaration and its subsequent revisions, the patients’ objective for participating in the study was described to them, and their informed consent was obtained prior starting treatment. NCT05371418 is the approved Clinical Trial Number.

### Criteria for teeth selection

#### A) Clinical criterion

(1) over-erupted tooth has angular bone loss with the presence of opposing; (2) mobility doesn’t exceed grade two; (3) no gingival recession more than 3 mm.

Exclusion criteria: (1) systemic diseases that make oral surgery more difficult, such as hyperparathyroidism, bleeding disorders, and so on (2). smokers’ patients (3). negative attitude toward oral hygiene.

#### Clinical procedures

**Fig. 1:**
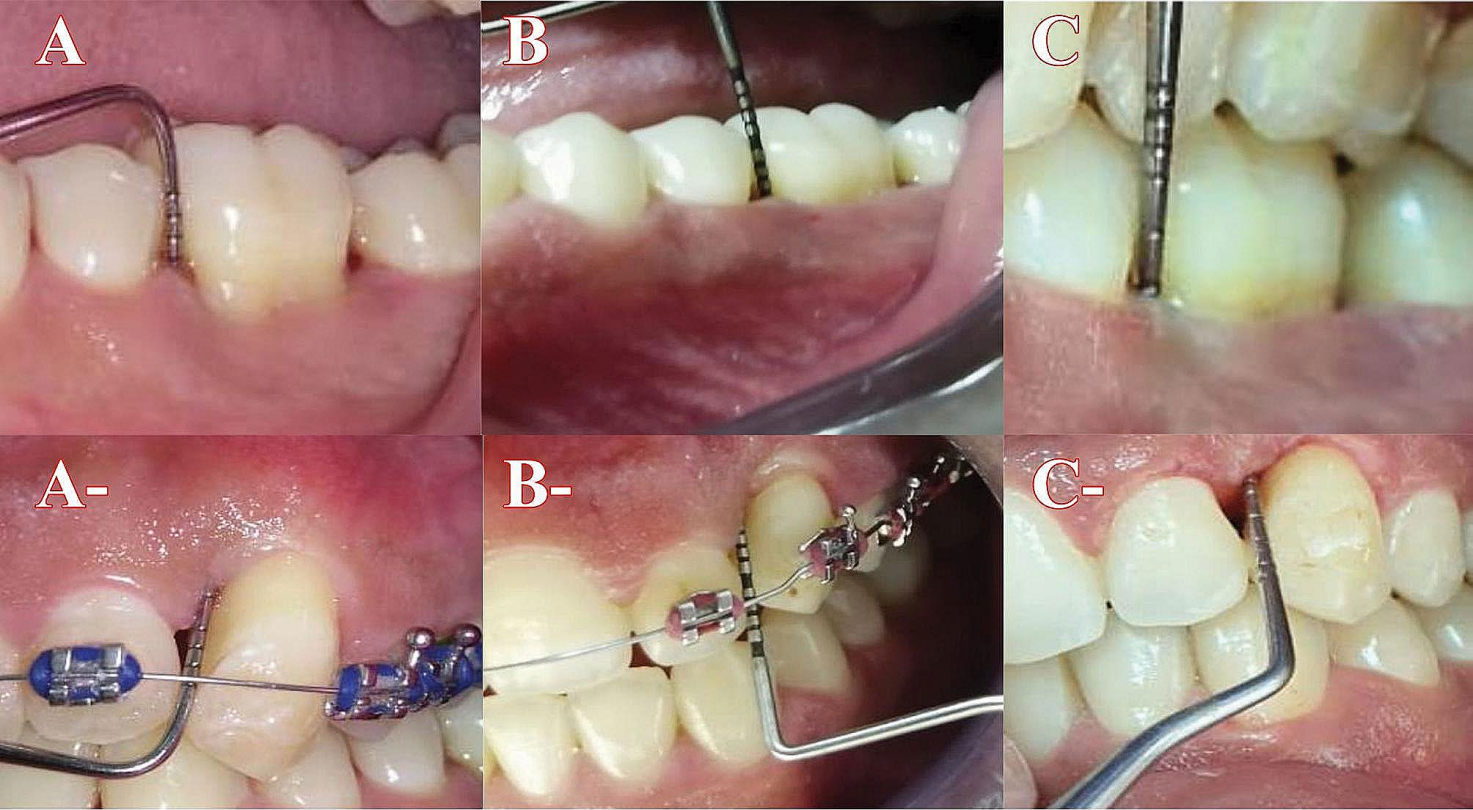
**A**, **B** and **C** represent pocket depth measurement at re-evaluation, six months after GTR and 6 months post orthodontic intrusion in G1 respectively, **A**-, **B**- and **C**- represent pocket depth measurement at re-evaluation, six months post orthodontic intrusion and 6 months after GTR in G2 respectively

Before beginning phase one, all patients were evaluated for pocket depth (PD) [17], plaque index (PI) [18]bleeding on probing (BOP) [19], and tooth mobility (TM) [20], as well as six and twelve months after beginning either GTR or OI as shown in Table (1) regarding clinical characteristic features of both groups and Fig. (1). In GTR, Bio-Oss bone graft (Geistlich PharmaAG, Wolfhusen, Switzerland, particle size 0.25–1 mm) and bovine collagen membrane (BioMend®, Sulzer Calcitek, Carlsbad, CA, USA) were used.

**Table 1:** Clinical characteristic features of G1 and G2

Clinical parameter assessed	For both groups
**Pocket depth (PD)**	PD was assessed from the gingival margin to the base of the pocket with PD in both groups greater than 5 and less than 8
**plaque index (PI)**	The measurement of the state of oral hygiene evaluated by the presence or absence of visible plaque present at the tooth margin with gingiva the following score: • Score 0: No plaque • Score 1: Plaque only recognized by disclosing tab or using a running probe • Score 2: moderate deposition of the plaque can be seen by the naked eye • Score 3: excess of soft matter within the gingival pocket and or on the gingival margin or tooth • With score 0 excellent, 0.1–0.9 good,1-1.9fiar, and 2–3 poor All patients revealed positive attitudes towards oral hygiene with being in the category of good oral hygiene at the re-evaluation phase
**Bleeding on probing (BOP)**	(BOP) was assed as; Score (0): Healthy gingiva; no bleeding upon insertion of periodontal probe interproximal Score (1): Edematous, reddened gingiva; no bleeding upon insertion of periodontal probe interproximal Score (2): Bleeding without flow upon periodontal probe interproximal Score (3): Bleeding with flow along gingival margin upon insertion of periodontal probe interproximal Score (4): Copious bleeding upon insertion of periodontal probe interproximal Score (5): Severe inflammation, marked redness with liability to spontaneous bleeding. All patients of both groups showed positive oral hygiene with a decreased mean value of BOP from the base to the re-evaluation phase
**Tooth mobility (TM)**	with giving score M0: Physiological mobility, M1: Slightly increased mobility, M2: Definitive considerable increase in mobility but no impairment of function, M3: Extreme mobility, a loose tooth that would be incompatible with function. All patients of groups selected with grade mobility from M0, M1, and M2 only. Cases of M3 mobility were completely excluded.

#### Grouping

The twenty chosen teeth from each of the ten patients were divided into two sets of ten randomly using sealed envelopes into.

- **Group1**: treated by GTR followed by OI.
- **Group 2**: treated by orthodontic intrusion followed by GTR.

As, in this split-mouth study, ten patients with at least two over-erupted teeth with the presence of their opposing were allocated into two groups. G1 was treated with GRT followed by OI, and G2 was treated by OI followed by GTR. All cases were treated in the periodontology and orthodontic clinics of the faculty of dentistry at Tanta University. All patients were evaluated clinically before starting phase one, at re-evaluation, 6 months after starting GTR or OI, and one year after the re-evaluation phase. Radiographical evaluation was made at the re-evaluation phase, 6 months after starting GTR or OI, and one year after the re-evaluation phase.

### Surgical procedure

**Fig. 2:**
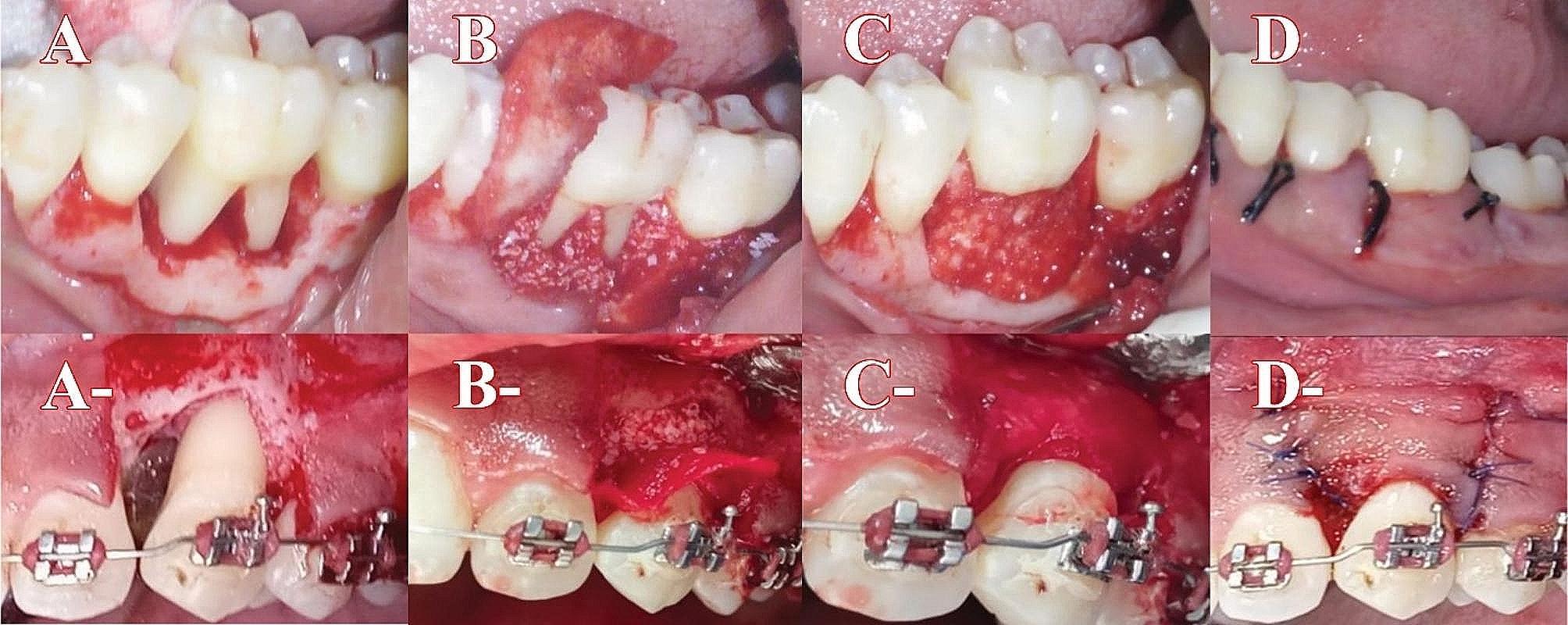
**A**, **B**, **C**, and **D** represent shape of the defect after full thickness flap reflection, bone graft and membrane application to the defect, complete membrane adaptation with complete coverage of bone graft, and flap repositioning with suturing in G1 respectively, **A**-, **B**-, **C**- and **D**-represent shape of defect after full thickness flap reflection, bone graft and membrane application to the defect, complete membrane adaptation with complete coverage of bone graft and flap repositioning with suturing in G2 respectively

Anesthesia was achieved by administering 2% lidocaine and 1:200,000 epinephrine. Intra-sulcular incision around the neck of the selected tooth and extending mesially and distally around adjacent teeth. One oblique releasing incision was made mesially, If the mesial vertical did not provide a clear view of the facial bone, a distal vertical incision would be made. To enable tension-free flap movement, a buccal full-thickness pyramidal flap was reflected beyond the mucogingival border. The granulation tissue was completely debrided from the defect, then the defect wall was thoroughly irrigated with saline. Bio-Oss bone graft was used to close the defect gap. The bone transplant and defect site were covered by a collagen membrane. A periodontal dressing was used after the gingival incision was closed with interrupted sutures as shown in (Fig. 2).

### Post-operative care

Each patient received directions on how to rinse with 0.1% chlorhexidine following surgery (twice daily for 2 weeks). A combination antibiotic therapy comprised of tablets containing Amoxicillin/Clavulanate 375 mg and Metronidazole 250 mg twice daily was also administered along with systemic anti-inflammatory medication for a week. In accordance with Horwitz’s guidelines [21]. The periodontal dressing and sutures were removed after 14 days.

### Orthodontic intrusion work after re-evaluation phase in G2 and six months post GTR in G1

Straight wire Roth devices were given to each subject (Ormco. USA, 0.0220.028 inch slot). Fixed appliance therapy was instituted until the initial phase of leveling and alignment was completed. According to individual case needs, sequential aligning arch wires were used until reaching a wire gauge of rectangular 0.016 × 0.022-inch stainless steel arch wire. Step-up and step-down bends were used to settle in the over-erupted teeth. In only one patient, the intrusive movement was carried out with the lower removable acrylic posterior bite plate for the intrusion of an over-erupted first molar [22]. The patients received hygiene instructions and planned to attend the orthodontic clinic for a regular recall visit (every 6 weeks).

#### B-Radiographic criteria

[1] Evidence of angular bone loss [2]. Absence of periapical radiolucency.

All patients selected for the study were submitted for periapical radiography in the selected region to ensure the presence of vertical bone loss and the absence of periapical radiolucency before starting phase one therapy using the same exposure parameters with 70 kVp, 6 mA, and 0.8 s exposure time.

Also, at the end of re-evaluation, at six and twelve months after starting either GTR or orthodontic intrusion, radiographic evaluation was assessed using CBCT scan to evaluate [1] The amount of orthodontic intrusion; [2] the amount of change in bone area; and [3] The amount of change in length related to angular defect in both groups using fixed exposure parameters of 5 mA and 120 kV, using the same region of interest in each case to ensure standardization. On Demand application software was used to make the images at the same axial slicing to obtain standardized coronal and sagittal images.

### Evaluation amount of orthodontic intrusion

**Fig. 3:**
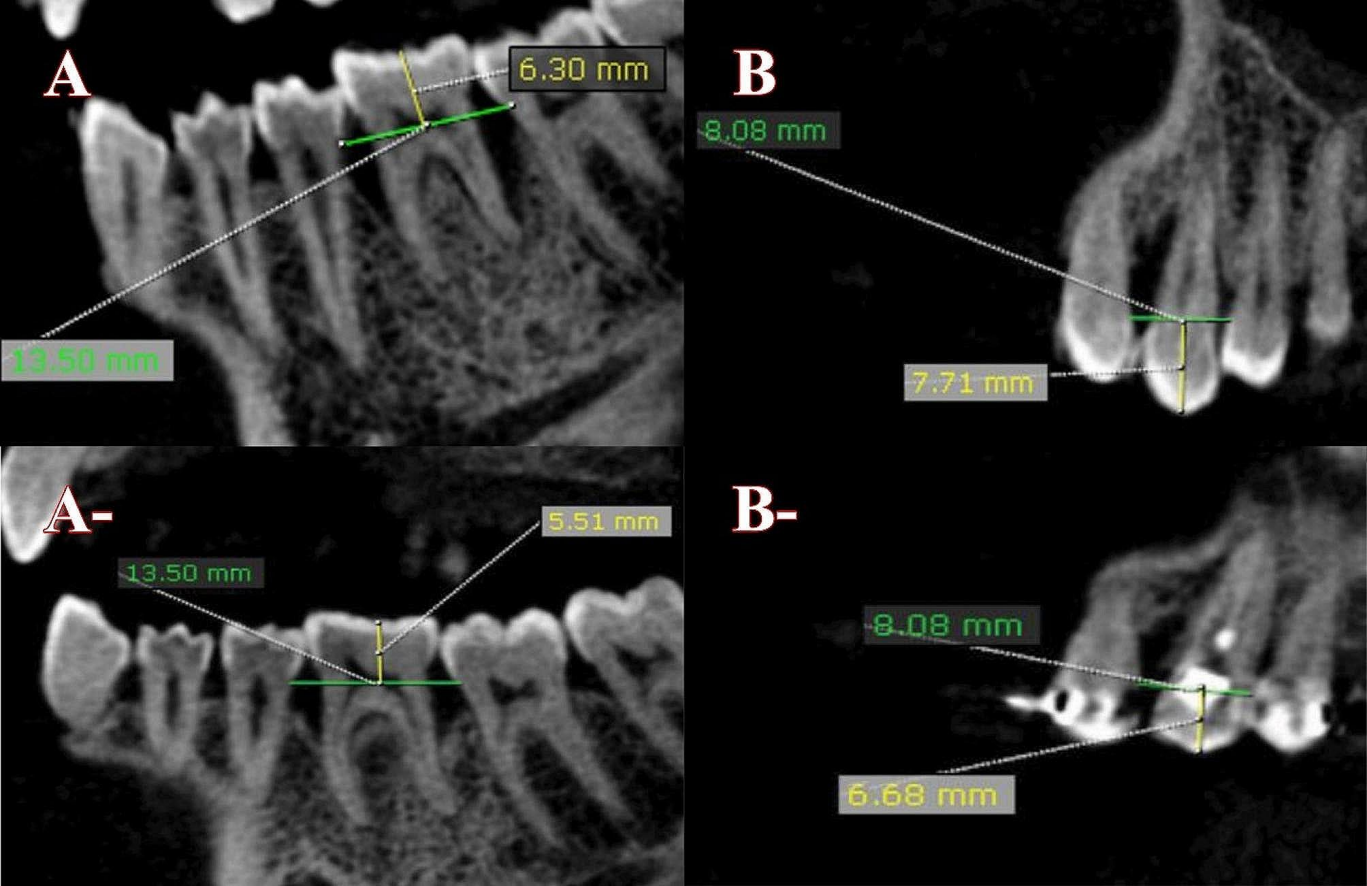
**A**, **A**-, represent the length of OIL at re-evaluation and 1 year after the re-evaluation phase of G1 respectively, **B**, **B**- represent the length of OIL at re-evaluation and 6 months after the re-evaluation phase of G2 respectively

The amount of orthodontic intrusion was assessed at the re-evaluation phase for both groups by using OnDemand application software to make a straight line from the cementoenamel junction (CEJ), referred to as the CEJ line, of both two teeth surrounding the over-erupted tooth, followed by a perpendicular line from the middle of the tip coronal margin of the over-erupted tooth reaching the CEJ line, and it was referred as the orthodontic intrusion line (OIL). Six months after the ending amount of orthodontic intrusion, which was one year after finishing the re-evaluation phase in G1 and nearly six months after the re-evaluation phase in G2, the same procedure was repeated using the same axial slicing to ensure standardization. Then the amount of orthodontic intrusion was equal to the length of OIL at 6 months after finishing OI, subtracted from OIL at the end of the re-evaluation phase as shown in (Fig. 3).

### Measurement amount of change in bone area related to the defect

**Fig. 4:**
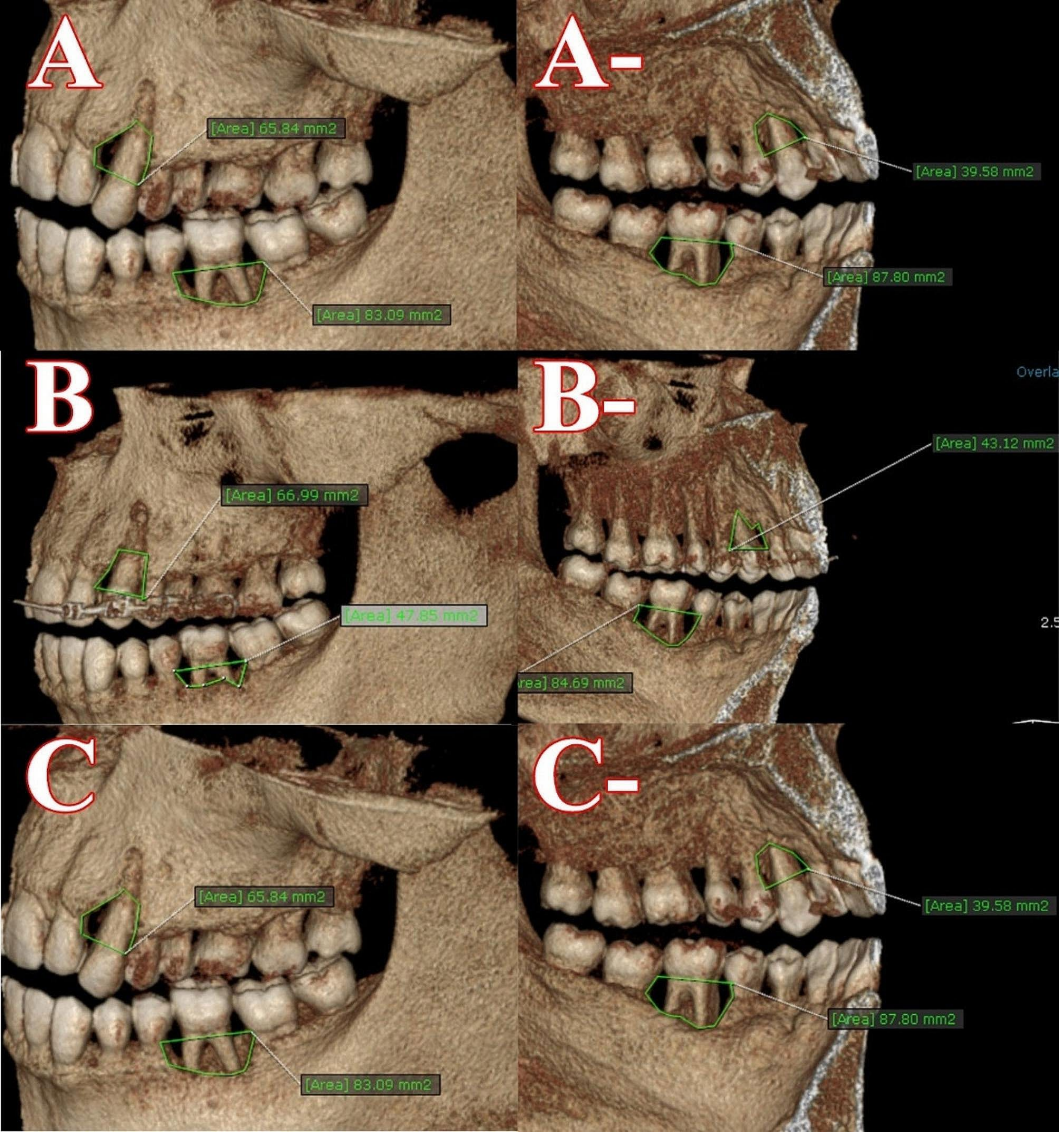
**A**, **A**-represent measurements of G1 and G2 defect areas at the re-evaluation phase from the facial and lingual regions, respectively. **B**, **B**-represent measurements of the defect area of G1 & G2 at 6 months after GTR for G1 and 6 months after OI for G2 from the facial and lingual regions respectively. **C**, **C**-represent measurements of G1 and G2 defect areas one year after re-evaluation from the facial and lingual regions, respectively

The bone area was measured using reconstructive 3D images of the lingual and facial regions from the lingual and facial views. The facial bone defect areas were measured for G1 and G2 by drawing a line from the CEJ of two teeth surrounding the over-erupted tooth and continuing the line to encircle the entire defect on the over-erupted tooth. The same was done to measure the area of the defect in the lingual region. These measurements were made at re-evaluation, 6 months after GTR for G1 or OI for G2, and 1 year after the end of the re-evaluation phase for both groups, as shown in (Fig. 4).

### Measurement of the defect depth

**Fig. 5:**
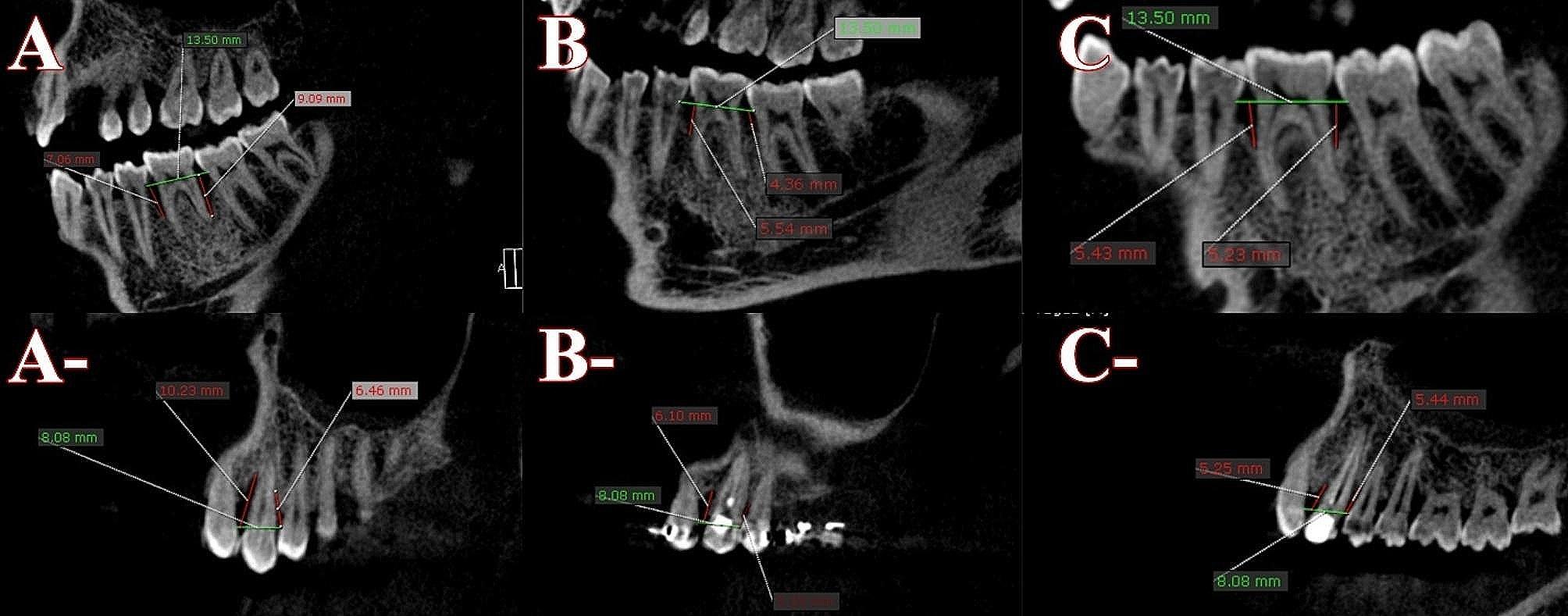
**A**, **B**, and **C** represent measurements of defects depth of G1 at the re-evaluation phase, 6 months after GRT and 1 year from re-evaluation respectively. **A**-, **B**- and **C**- represent measurements of defects depth of G1 at the re-evaluation phase, 6 months after OI and 1 year from re-evaluation respectively

The defect depth was measured by drawing a CEJ line between both teeth surrounding the over-erupted tooth, followed by two lines perpendicular to the CEJ line from the distal and mesial sides of the over-erupted tooth to the deepest point of the defect. These measurements were done using the same axial slicing to ensure standardization at re-evaluation, 6 months after GTR for G1 or OI for G2, and 1 year after the end of the re-evaluation phase for both groups, as shown in Fig. 5.

### Statistical analysis

All data was gathered, collated, and statistically evaluated. The significance of connections between nominal variables was examined using the chi-square test and the Wilcoxon test. The paired-t test was utilized to compare the means of the groups. *P* value ≤ 0.05 was used as the significance threshold. SPSS version 16 was used to conduct the statistical analysis.

## Results

### Clinical evaluation

Before starting treatment and at the reevaluation phase, the G1 group revealed 1 tooth (10%) with grade M0 mobility, 7 teeth (70%) with grade M1 mobility, and 2 teeth (20%) with grade M2 mobility, whereas the G2 revealed 2 teeth (20%) with grade M0 mobility, 6 (60%) teeth with grade M1 mobility, and 2 (20%) teeth with grade M2 mobility. The Chi-square test revealed no statistically significant difference between the two groups (*p* > 0.05). While six months post-GTR, G1 revealed 3 teeth (30%) with grade M0 mobility, 6 teeth (60%) with grade M1 mobility, and 1 tooth (10%) with grade M2, whereas G2 six months post-OI revealed 1 tooth (10%) with grade M0 mobility, 4 teeth (40%) with grade M1 mobility, and 5 (5% of the total) teeth with grade M2 mobility with using the Chi-square test, there was a statistically significant difference (*P* value ≤ 0.05) in favor of G1. In addition, one year after re-evaluation, G1 showed 2 teeth (20%) with grade M0, 6 teeth (60%) with grade M1 mobility, and 2 teeth (20%) with grade M2 mobility, whereas G2 revealed 5 teeth (50%) with M0 mobility, 4 teeth (40%) with grade M1 mobility, and 1 tooth (10%) with grade M2 mobility. By using the chi-square test, there was a statistically significant (*P* value ≤ 0.05) in favor of G2, as shown in Table (2).

**Table 2:** Inter-group comparison of mobility between G1 and G2 before treatment, at the re-evaluation phase, six months post-GTR or OI, and one year after re-evaluation

**G1 at PT** ^**α**^ ** and Re** ^**∞**^						**G1 at 6** ^**∂**^						**G1at 12** ^**¥**^					
M0		M1		M2		M0		M1		M2		M0		M1		M2	
*N*	%	*N*	%	*N*	%	*N*	%	*N*	%	*N*	%	*N*	%	*N*	%	*N*	%
1	10%	7	70%	2	20%	3	30%	6	60%	1	10%	2	20%	6	60%	2	20%
**G2 at PT** ^**α**^ **and Re** ^∞^						**G2 at 6** ^∂^						**G2at 12** ^¥^					
M0		M1		M2		M0		M1		M2		M0		M1		M2	
*N*	%	*N*	%	*N*	%	*N*	%	*N*	%	*N*	%	*N*	%	*N*	%	*N*	%
2	20%	6	60%	2	20%	1	10%	4	40%	5	50%	5	50%	4	40%	1	10%
G1V G2 at PT and re-evaluation						G1 at 6monthspost GTR V G2 at 6monthspostOI						G1V G2 at one year after re-evaluation					
**χ2**			***P***			**χ2**			***p***			**χ2**			***P***		
4.103			0.1286ns			40.67			0.0001***			10.91			0.0043**		

Chi-square test Significance: p*<0.05, p**<0.01, p***<0.001; ns = not significance; α before the treatment, ∞ at re-evaluation phase, ∂at 6 months post-GTR (G1) or OI(G2) and¥ at one year from re-evaluation.

The plaque index (PI) was assessed by measuring the amount of plaque in the mesial, distal, buccal, and lingual surfaces of the over-erupted tooth. These values were totaled and divided by four to measure the mean of the plaque index for each over-erupted tooth. Before treatment, the mean PI values for groups were 1.875 and 2.025 for G1 and G2 respectively, whereas at the reevaluation phase, the mean PI values were 0.7750 and 0.6250 for G1 and G2, respectively. The mean PI values at 6 months after GTR or OI were 0.4600 and 0.75 for G1 and G2 respectively, and 0.6250, and 0.7750 at one year after re-evaluation for G1 and G2 respectively. The results of the Wilcoxon matched pairs test, revealed no statistically significant differences (*p* > 0.05) between the two groups across all stages, as shown in Table (3).

**Table 3:** Inter-group compression of the mean values of plaque index between G1 and G2

	G1vG2 at PT ^α^	G1vG2 at Re^∞^	G1vG2 at 6^∂^	G1vG2 at 12^¥^
MeanG1	1.875	0.7750	0.4600	0.6250
MeanG2	2.025	0.6750	0.6250	0.7750
Z	-12.00	11.00	-22.00	-22.00
*P*	0.3482ns	0.5450ns	0.0655ns	0.1236ns

Wilcoxon matched-pairs signed rank test: p*<0.05, p**<0.01, p***<0.001; ns = not significance; α prior treatment, ∞ at re-evaluation phase, ∂at 6 months post-GTR (G1) or OI(G2) and^¥^ at one year from re-evaluation

The bleeding on probing (BOP) was assessed by measuring the values of mesial and distal papillae related to the over-erupted tooth from the facial and lingual sides, and then these values were totaled and divided by 4 to measure the mean of the papillary bleeding index for each over-erupted tooth. Before treatment, the mean BOP values for groups 1 and 2 were 2.5 and 2.9, respectively, whereas at the reevaluation phase, the mean BOP values were 0.825 and 0.8 for G1 and G2, respectively. The mean BOP values at 6 months after GTR or OI were 0.6000 and 0.75 for G1 and G2 respectively, and 0.8 at one year after re-evaluation for both groups. The results of the Wilcoxon matched pairs test, revealed no statistically significant differences (*p* > 0.05) between the two groups across all stages, as shown in Table (4).

**Table 4:** Inter-group compression of the mean values of bleeding on probing between G1 and G2

	G1vG2 at PT ^α^	G1vG2 at Re^∞^	G1vG2 at 6^∂^	G1vG2 at 12^¥^
MeanG1	2.500	0.8250	0.6000	0.8
MeanG2	2.900	0.8000	0.7500	0.8
Z	-24.00	3.000	-12.00	-1.000
*P*	0.1052ns	0.9179ns	0.4332ns	1ns

Wilcoxon matched-pairs signed rank test: p*<0.05, p**<0.01, p***<0.001; ns = not significance; α before the treatment, ∞ at re-evaluation phase, ∂at 6 months post-GTR (G1) or OI (G2) and^¥^ at one year from re-evaluation.

Pocket depth (PD) was assessed by measuring the values of mesial line angle, distal line angle, and mid-line angle from the facial and lingual regions of the over-erupted tooth and totaling them, then dividing by 6. Before the treatment, the mean values of PD were 6.590 ± 0.5259 and 6.420 ± 0.5224 for G1 and G2 respectively. Upon a paired t-test, there were no statistically significant differences (*p* > 0.05) before the treatment and at the re-evaluation phase. Whereas, at 6 months post GTR or OI, the mean values of PD were 3.330 ± 0.4373&7.230 ± 0.5187 for G1 and G2respectively, with a paired t-test, there was a statistically significant difference (*P* ≤ 0.05) in favor of G1. However, one year after re-evaluation, the mean values of PD were 6.250 ± 0.4453 and3.660 ± 0.4600 for G1 and G2 respectively. With a paired t-test, there was a statistically significant difference (*P* ≤ 0.05) in favor of G2, as shown in Table (5).

**Table 5:** Inter-group compression of mean values of pocket depth between G1 and G2

	G1vG2 at PT ^α^	G1vG2 at Re^∞^	G1vG2 at 6^∂^	G1vG2 at 12^¥^
Mean ± SDG1	7.000 ± 0.5270	6.590 ± 0.5259	3.330 ± 0.4373	6.250 ± 0.4453
Mean ± SDG2	7.000 ± 0.5099	6.420 ± 0.5224	7.230 ± 0.5187	3.660 ± 0.4600
t	0	0.8361	23.49	12.24
*P*	1ns	0.4248ns	0.0001***	0.0001***

paired t-test: p*<0.05, p**<0.01, p***<0.001; ns = not significance; α before the treatment, ∞ at re-evaluation phase, ∂at 6 months post-GTR (G1) or OI(G2), and^¥^ at one year from re-evaluation

### Radiographical evaluation

Intergroup assessment of the mean values of the amount of orthodontic intrusion were1.141 ± 0.2313 and1.090 ± 0.2601 for G1 and G2 respectively. Upon a paired t-test, there was no statistically significant variation (*p* > 0.05) between the two groups as shown in Table (6).

**Table 6:** Inter-group comparison of mean values of orthodontic intrusion between G1 and G2

group	G1^¥^	G2^∂^	
M ± SD	1.141 ± 0.2313	1.090 ± 0.2601	
t	0.4145		
*P*	0.6882		

Paired t test Significance: p*<0.05, p**<0.01, p***<0.001; ∂at 6 months post OI(G2) and^¥^ at one year from re-evaluation.

At the re-evaluation phase, 6 months post-GTR for G1 and 6 months post-OI for G2, the amount of defect depth was assessed for each over-erupted tooth by adding the defect depth from the mesial and distal sides to the over-erupted tooth and dividing by 2. The mean values of defect depth at the re-evaluation phase were 7.410 ± 0.5466 and 7.390 ± 0.5840 for G1 and G2 respectively, whereas at 6 months post-GTR or OI were 4.540 ± 0.4949 and 5.720 ± 0.8189 for G1 and G2 respectively, and the mean values of defect depth at one year from the re-evaluation phase were 5.190 ± 0.4630 and 3.980 ± 0.5865 for G1 and G2 respectively. Regarding using a paired t-test, there was no statistically significant variation (*p* > 0.05) between the two groups at re-evaluation, but at 6 months post GTR or OI, there was a statistically significant difference (*P* ≤ 0.05) in favor of G1. However, after one year after the re-evaluation, G2 had a statistically significant advantage (*P* value ≤ 0.05), as shown in Table (7).

**Table 7:** Inter-group comparison of mean values of defect depth between G1 and G2

	G1v G2 at Re^∞^	G1v G2 at6^∂^	G1v G2 at12^¥^
M ± SD	7.410 ± 0.5466	4.540 ± 0.4949	5.190 ± 0.4630
	7.390 ± 0.5840	5.720 ± 0.8189	3.980 ± 0.5865
t	0.4286	3.915	6.111
*P*	0.6783ns	0.0035***	0.0002***

The amount of defect area was assessed for each over-erupted tooth by summing the defect area from the buccal and facial sides of the over-erupted tooth and dividing by 2 at the re-evaluation phase, 6 months post GTR for G1 and 6 months post OI for G2. The mean values of defect area at the re-evaluation phase were 72.57 ± 11.16 and 73.94 ± 10.01for G1and G2 respectively, whereas at 6 months post GTR or OI were 60.37 ± 9.771and76.29 ± 9.842 for G1and G2 respectively, and the mean values of defect depth at one year from the re-evaluation phase were 70.83 ± 10.77and 61.08 ± 10.49 for G1 and G2 respectively.  A paired t-test indicated no statistically significant differences (*p* > 0.05) between the two groups during the re-evaluation phase and one year after the re-evaluation phase, but there was a statistically significant difference (*P* ≤ 0.05) in favor of G1 at 6 months post GTR or OI, as shown in Table (8).

**Table 8:** Inter-group comparison of mean values of bone defect between G1 and G2

	G1v G2 at Re^∞^	G1v G2 at6^∂^	G1v G2 at1^¥^
M ± SD	72.57 ± 11.16	60.37 ± 9.771	70.83 ± 10.77
	73.94 ± 10.01	76.29 ± 9.842	61.08 ± 10.49
t	0.2404	3.287	1.732
*P*	0.8154ns	0.0094**	0.1172ns

Paired t-test Significance: p*<0.05, p**<0.01, p***<0.001; α before the treatment, ∞ at re-evaluation phase, ∂at 6 months post-GTR (G1) or OI(G2) and ¥ at one year from re-evaluation

Measurements of dimensional changes of bone area were assessed in G1 and G2 by subtracting the value of bone area at 6 months post GTR or OI from the bone area at Re-evaluation (G1Re° -G16^∂^) and (G2Re° -G26^∂^) for G1 and G2 respectively, subtracting value of bone area after one year after re-evaluation phase from re-evaluation phase (G1Re° -G12^¥^), (G2Re° -G212^¥^) respectively and subtracting bone area at one year after re-evaluation from bone area at 6 months post GTR or OI (G16^∂^- G112^¥^),(G26^∂^- G212^¥^) for G1 and G2 respectively. The mean values of (G1Re° -G16^∂^) and (G2Re° -G26^∂)^ were 12.50 ± 4.605 and − 2.084 ± 0.9213 respectively. Upon a paired t-test, there was a statistically significant variation (*P* ≤ 0.05) in favor of G1, the mean values of (G1Re° -G12^¥^) and (G2Re° -G212^¥^) were 2.038 ± 1.685 and12.43 ± 3.066 by using a paired test, there was a statically significant variation (*P* ≤ 0.05) in favor of G2. The mean values of (G16^∂^- G112^¥^) and (G26^∂^- G212^¥^) were-10.46 ± 3.563 and14.51 ± 3.247 with using a paired t-test there was a statically significant difference (*P* ≤ 0.05) in favor of G2 in the Table (9).

**Table 9:** Inter-group comparison of the mean values of area dimensional changes between G1 and G2

	G1Re° -G16^∂^ v G2Re° -G26^∂^	G1B-G112^¥^ v G2B -G212^¥^	G16^∂^- G112^¥^ v G2^∂^”- G212^¥^
M ± SD	12.50 ± 4.605	2.038 ± 1.685	-10.46 ± 3.563
	-2.084 ± 0.9213	12.43 ± 3.066	14.51 ± 3.247
t	6.163	5.933	11.23
*p*	0.0035**	0.0040**	0.0004***

Paired t-test Significance: p*<0.05, p**<0.01, p***<0.001, ∞reevaluation phase, ∂six months after (GTR G1 or orthodontic intrusionG2), _¥_ one year from re-evaluation

## Discussion

Pathological tooth migration is commonly associated with moderate to severe periodontitis, especially in the upper anterior region [4, 9]. This disease process enhances the infra-bony defects with accompanying incisal and facial displacements [23]. Histological evidence suggests that orthodontic intrusive movement may be beneficial for that condition if good oral hygiene is maintained [24]. According to the hypothesis, intrusion can positively affect the involved tooth periodontium; the stretched periodontal ligament fibers prevent the downward growth of epithelial cells while stimulating the peri-radicular cellular activity, hence the new cementum and connective tissue attachment formation [25–27] The studies reveal a great deal of controversy among the proponents of the treatment sequence, some investigators [28, 29] insist that defect regeneration should precede orthodontic movement, while others [30, 31] resorted to orthodontic treatment first. Therefore, the present controlled clinical trial was designed to test the hypothesis that one strategy for over-erupted tooth with angular bone loss (GTR followed by OI or OI followed by GTR) would be superior to the other one.

Periodontal therapy is considered necessary in teeth with occlusal abnormalities because it is known that orthodontic intrusion of over-erupted teeth with angular bone loss is effective if done in a healthy periodontium, leading to an improvement in the periodontium’s condition by changing the defect dimension [24, 32]. However, if the periodontal defects are severe enough to prevent the patient from performing proper oral care, GTR must begin before orthodontic intrusion [23].

Due to the presence of deep pockets, our patients were subjected to performing proper oral hygiene, resulting in an obvious reeducation of the mean values of bleeding on probing (POP) from base values of 2.5 and 2.9 for G1 and G2, respectively, to 0.825 and 0.8 at the re-evaluation phase for G1 and G2, respectively, with nearly the same mean value at one year from re-evaluation (0.8) for both groups to ensure proper periodontal health during treatment.

It should take into consideration the difficulty of comparing these research results with previously published studies as, according to our knowledge there was a limited number of studies on traumatic occlusion, which led to unfavorable tooth position and bone loss. Furthermore, fewer studies have addressed the issue of bone loss in periodontally involved patients, which leads to tooth movement, indicating the need for orthodontic correction with periodontal therapy.

This study unveiled an obvious significant decrease in TM and PD six months after re-evaluation in favor of G1 when compared with G2, with a statistically significant decrease in TM and PD in favor of G2 when compared with G1, which means bone loss after a bone gain in GI. The transient improvement in G1 was due to the osteoconductive and excellent properties of the bio-oss bone graft [33–35] in GTR; however, in G1, the tooth was still in an unfavorable position, leading to the persistence of traumatic occlusion and further bone loss, particularly after 6 months from GTR (the start of OI) [36].

Our clinical results discovered a statistically significant (*P* ≤ 0.05) decrease in PD and TM in favor of G2 one year after the re-evaluation phase, which is consonant with Tietmann et al. [37] who conducted a study to evaluate the efficacy of GTR followed by orthodontic movement 3 months after GTR in the treatment of 526 intra-bony defects in 48 patients with stage IV periodontitis.

Due to better viewing of defect dimensions in 3-D planes, which provides multiple information guiding us for a proper and suitable treatment plan for the cases, CBCT is considered a revolutionary innovation in x-ray imaging modality over conventional x-ray imaging modality [38–40].

After 6 months of re-evaluation, our radiographic results revealed a statistically significant (*P* ≤ 0.05) decrease in defect depth and defect area dimensional changes in favor of G1. This could be due to the presence of periodontal inflammation in the G2 group after sifting supra-gingival plaque into sub-gingival plaque with the presence of a deep periodontal pocket, which was not noticed during the months between follow-up visits because patients may have neglected very strictly required oral hygiene methods, and this inflammation would lead to bone loss with orthodontic intrusion.

Our results agreed with an experimental study on dogs performed by Ericsson et al.,^(32)^ by making artificial periodontal pockets related to fourth per-molars after extraction 3rd premolars on five dogs. These premolars were exposed to tipping orthodontic occlusal with allowing brushing twice of control groups and tests did not perform any oral hygiene. On sacrificing these dogs, the histological studies revealed the plaque-infected teeth with orthodontic tipping force resulted in the formation of infrabony pockets whereas when comparable orthodontic forces were applied to teeth that were free of plaque, infrabony pockets were not created.

This research also revealed an obvious significant decrease (*P* value ≤ 0.05) in defect depth and defect area dimensional changes one year after the re-evaluation phase in favor of G2. Although G1 showed better defect dimensional improvement at six months from re-evaluation but there was a deterioration regarding defect dimensional area which was increased at one year from the re-evaluation phase. These results could be due to the presence of the teeth in the G1 group after six months from GTR in an abnormal occlusal position leading to the persistence of traumatic occlusion which could lead to bone loss before reaching a suitable comforting position suitable for proper periodontal heath out of traumatic occlusion.

These results were confirmed by Nunn and Harrel [41] whose study aimed to study the influence of traumatic occlusion on periodontally involved patient’s occlusion who were split up into three categories. The first group got no treatment, the second group only underwent surgical treatment, and the third group received the necessary ideal treatment. These patients were evaluated for pocket depth concerning occlusal disturbance which revealed patients with occlusal disturbance had deeper pockets and worse teeth mobility with poor prognosis than patients with no occlusal discrepancies suggesting occlusal disharmony had an impact role to be a risk factor for periodontal bone loss.

## Conclusion

Treatment of angular defect with an over-erupted tooth with the presence of opposing is considered a challenge whether to start guided tissue regeneration or orthodontic intrusion first. The treatment of angular bone loss with over erupted tooth is best treated even with deeper periodontal pockets by OI followed by GTR as OI makes the tooth in better periodontal heath out of occlusal trauma favoring the success of followed GTR.

### Limitation

This study was limited by being a controlled clinical trial with few case numbers due to the rarity of bilateral affection of angular bone loss with over-erupted teeth. Also, it is recommended to do the same study but with longer follow-up periods to evaluate the efficacy of GTR-followed OI in the long run term.

## Data Availability

All data are available from the corresponding author upon a reasonable request.
